# Pneumopericardium should be considered with electrocardiogram changes after blunt chest trauma: a case report

**DOI:** 10.1186/1752-1947-2-100

**Published:** 2008-04-04

**Authors:** Arjan JM Konijn, Peter HM Egbers, Michaël A Kuiper

**Affiliations:** 1 Department of Intensive Care, Medical Centre Leeuwarden, 8901 BR, Leeuwarden, The Netherlands; 2 Department of Intensive Care, Academic Medical Centre, 1100 DD, Amsterdam, The Netherlands

## Abstract

**Introduction:**

Electrocardiogram (ECG) abnormalities in patients with blunt chest trauma are diverse and non-specific, but may be indicative of potentially life-threatening conditions.

**Case presentation:**

We report a rare case of pneumopericardium with extreme ECG abnormalities after blunt chest trauma in a 22-year-old male. The diagnosis was confirmed using computed tomography (CT) scanning. The case is discussed, together with its differential diagnosis and the aetiology of pneumopericardium and tension pneumopericardium.

**Conclusion:**

Pneumopericardium should be distinguished from other pathologies such as myocardial contusion and myocardial infarction because of the possible development of tension pneumopericardium. Early CT scanning is important in the evaluation of blunt chest trauma.

## Introduction

When an electrocardiogram (ECG) is obtained during the diagnostic processing and evaluation of a trauma patient (as in the present case), it is important to realize that ECG findings in patients with cardiac trauma are diverse and non-specific. These findings may be non-specific ST-segment or T-wave changes, axis deviation and dysrhythmias, such as premature atrial contractions, bundle branch blocks and ventricular fibrillation [1]. Diagnostic considerations in a patient with blunt chest trauma and ECG abnormalities include, amongst others, myocardial contusion and myocardial ischaemia. Other causes involve the presence of air in thoracic structures that do not normally contain air, for example pneumothorax, pneumomediastinum and pneumopericardium. These options are discussed in a stepwise manner and related to the patient in this case report.

## Case presentation

A 22-year-old male, with no previous medical history, was admitted to the intensive care unit (ICU) at our hospital with blunt thoracic trauma and near-drowning after a high-energy trauma. The man had been driving a car when, for no apparent reason, he lost control and drove into a ditch filled with water.

**Figure 1:**
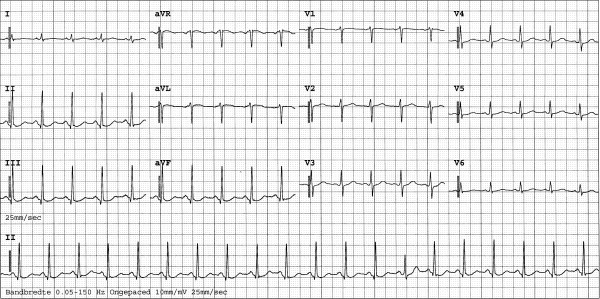
ECG performed on admission.

**Figure 2:**
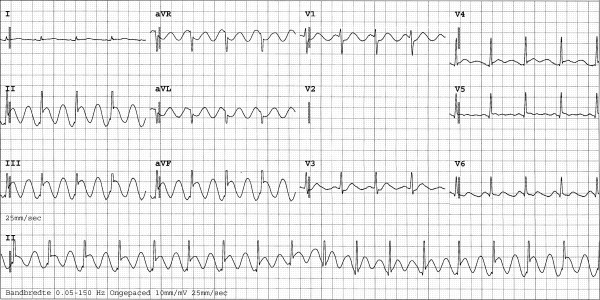
**ECG showing the most striking abnormalities.** Interestingly, there is no change in QRS amplitude, frequently seen in pericardial tamponade. Owing to technical problems, lead V2 is absent.

**Figure 3:**
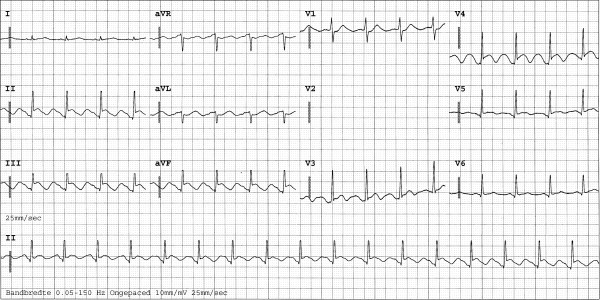
**ECG performed shortly after drainage.** The remaining abnormalities resolved completely in approximately 12 h. Owing to technical problems, lead V2 is absent.

**Figure 4:**
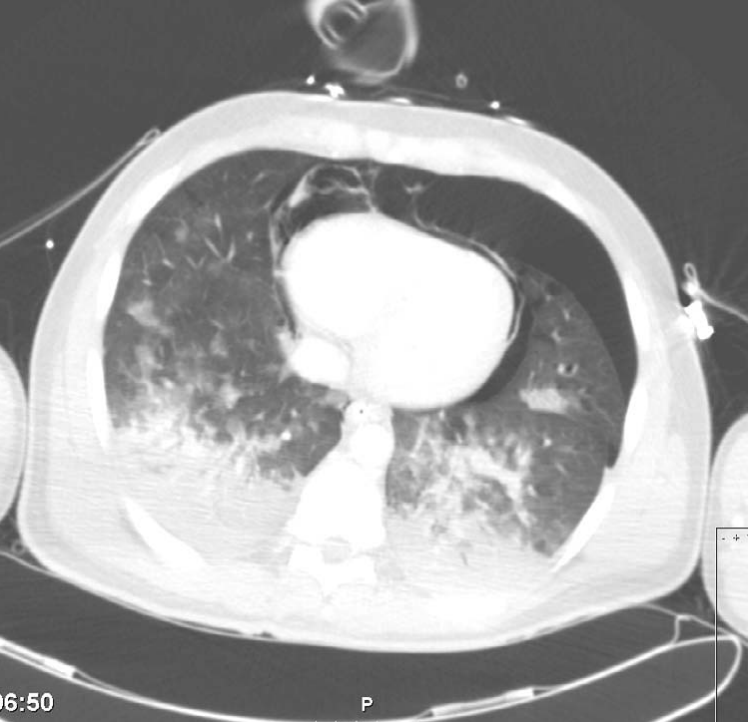
**CT scan, transversal view, showing pneumothorax, pneumomediastinum and pneumopericardium.** Haematothorax is present at the time of scanning.

The patient consequently aspirated water, but managed to reach solid ground. He was transported by ambulance to the hospital emergency unit, where he was found to be in respiratory failure, probably as a result of severe lung contusion. He was subsequently intubated and mechanically ventilated. During the first few days of admission, pressure-controlled ventilation was used with relatively high ventilator settings. During the first hours after admission these settings were a positive end-expiratory pressure level of 18 cm H_2_O, inspiratory pressure level of 13 cm H_2_O, a fractional inspired oxygen level of 60% and a respiration frequency of 30 cycles per minute. No recruitment manoeuvre was performed. A central venous line was inserted into the right femoral vein. Other than fractures of the left clavicle and superficial haematomas, there were no abnormalities on physical examination of the thorax. In particular, no asymmetrical pulmonary auscultation, subcutaneous emphysema or abnormal heart sounds were present. Chest radiography and a 12-lead ECG were performed on admission (Figure 1). No significant abnormalities were observed at the time, but shortly thereafter the ECG showed ST-depression in leads II, III, aVF and V3 and V4. No major haemodynamic problems occurred, and the creatine phosphokinase and accessory MB-fraction indicated only a loss of skeletal muscle. However, extreme ECG abnormalities developed during the following hours (Figure 2 and 3). CT scanning of the thorax, performed approximately 12 hours after admission, showed a pneumopericardium, as well as pneumomediastinum and bilateral pneumothorax (Figure 4). Severe lung contusion and haematothorax were also apparent on these images.

Transoesophageal echocardiography (TEE) was performed after transthoracic echocardiography had failed to deliver the required image quality. TEE did not identify any wall motion abnormalities, and the accident appeared to have had no abdominal or cerebral repercussions.

Both the pneumothorax and pneumopericardium resolved after the insertion of a left-sided chest tube. The right-sided pneumothorax resolved spontaneously, after which the patient made a rapid recovery and was discharged from the ICU on day 5. He was discharged from hospital five days later, having made a complete recovery.

## Discussion

In this case, a likely differential diagnosis was myocardial contusion, which has a broad variety of presenting symptoms, the most frequent being precordial pain which is not relieved by analgesia. In addition to ECG changes, other findings include dyspnoea, pericardial friction rub, pulmonary rales and an elevated central venous pressure. This complex of symptoms may mimic those of acute coronary syndrome, although symptoms may also be completely absent. Myocardial contusion can be diagnosed using echocardiography, as this imaging modality visualizes the actual contusion as well as changes in cardiac chamber size, wall motion abnormalities and the presence of cardiac tamponade [2]. Echocardiography was performed on this patient after pneumopericardium had been diagnosed. Although cardiac contusion might easily have coexisted, none of the aforementioned abnormalities were seen. In such a situation it is important to recognize the inferior diagnostic quality of transthoracic echocardiography compared with TEE.

Acute coronary syndrome was unlikely to occur in this patient because he was young and had no predisposing medical history, such as angina [1]. However, even in young people traumatic myocardial infarctions have been reported that can result from acute thrombotic coronary occlusion, intimal tears and vessel rupture [3]. As mentioned above, the cardiac enzyme profile indicated a loss of skeletal muscle, with serial measurements of CK and accessory MB-fraction showing peak levels of 2,600 and 29 U/l, respectively. Unfortunately, the level of troponins, which has been shown to be more useful in detecting myocardial injury than CK and CKMB over the past decade, was not measured [4]. Nonetheless, it was concluded that significant myocardial contusion or infarction was highly unlikely.

The presence of extraluminal air is a frequent complication in cases of blunt thoracic trauma, because the differing electrophysiological behaviour of air can cause the ECG to change frequently. The incidence of pneumothorax in this population is approximately 40%, while that of pneumomediastinum may be as high as 10% (see [5,6]). Pneumopericardium, however, is rare and, to the best of the authors' knowledge, no incidence rates have been recorded. Neither have any clinical trials been conducted on trauma patients in which this pathological entity is described.

Traumatic rupture or penetration of the alveoli, pleurae and/or thoracic wall by fractured ribs may result in pneumothorax. In addition, tracheobronchial tears may cause pneumothorax, as well as pneumomediastinum, although this depends on the localisation of the lesion with respect to the position of the pulmonary ligament. Pneumothorax or pneumomediastinum occurs when the lesion lies distal or medial, respectively, to the pulmonary ligament. Other conditions that may lead to pneumomediastinum include oesophageal disruption and direct communication of the mediastinum with the pneumothorax. However, in the majority of cases pneumomediastinum results from alveolar rupture and/or positive-pressure mechanical ventilation. Initially, air leaks from the lumen of the lung and then travels along the peribronchovascular sheaths, dissecting in a medial direction and resulting in mediastinal air. This mechanism, which is known as the Macklin effect, was first described more than 65 years ago [6,7]. In the event of pneumopericardium, the Macklin effect is once again the major cause, although higher intrathoracic pressures are required; however, as these conditions share aetiology, it is not surprising that high intrathoracic pressures are often accompanied by pneumomediastinum. It is most likely that air enters the pericardial sac along the venous sheaths, where the collagenous support of the pericardial reflections is weaker [8]. Understandably, it may take some time for a clinically relevant pneumopericardium or pneumomediastinum to be revealed. The pericardial space may also be connected directly to pleural or tracheobronchial gases as a consequence of pericardial tear.

Brander et al. [9] reviewed previous reports on pneumopericardium which described symptoms such as chest pain, dyspnoea, palpitations, distant heart sounds, shifting precordial tympani, mill wheel murmur and different ECG findings such as ST depression/elevation, T-wave inversion and low voltages. However, as these authors stated, none of these was specific [9].

Options for the diagnosis of pneumopericardium, pneumothorax and pneumomediastinum include plain chest radiography, ultrasound and CT scanning. In trauma patients (such as the case reported here), CT scanning is the most appropriate imaging modality. Previous reports have described pneumopericardium in very diverse circumstances such as laparoscopy, in fistula formation between the oesophagus or bronchus owing to cancer or ulceration, in barotrauma in women who are in labour or during delivery, and in purulent pericarditis [9]. Imaging modalities other than CT scanning may be more appropriate, depending on the individual case.

Pneumopericardium is usually self-limiting and resolves spontaneously, but may require intervention such as drainage of the accompanying pneumothorax. Notably, complications such as tension pneumopericardium are described in up to 37% of reported cases. In this situation a pressurized compartment is created by the one-way valve principle, and possibly worsened by mechanical ventilation. This in turn may lead to a life-threatening cardiac tamponade, requiring emergency pericardiocentesis or surgery [10].

In the reported patient, the ECG changes occurred after emergency department evaluation and ICU admittance. No abnormalities were seen on plain chest radiography taken on admission, while CT scanning revealed pneumothorax, pneumomediastinum and pneumopericardium. These three entities, but predominantly pneumopericardium, are the most likely explanation for the extreme ECG changes. Pneumopericardium and pneumomediastinum most likely occurred at the trauma and worsened during mechanical ventilation, as the ECG abnormalities became more impressive as time progressed, and high-pressure mechanical ventilation was used. There was no indication for the presence of tension pneumopericardium, as no major haemodynamic problems had occurred. Drainage of the pneumothorax led to a resolution of the pneumopericardium and pneumomediastinum, which in turn resulted in a rapid normalisation of the ECG.

High-energy blunt chest trauma with bone fractures should heighten the suspicion of intrathoracic organ lesions. CT scanning is the preferred imaging modality in trauma patients, and should be performed at an early stage to exclude pneumothorax, pneumomediastinum, pneumopericardium, haematothorax and lesions of any intrathoracic structures such as aortic dissection. However, as many of these entities may develop and become clinically relevant within a few hours of the initial trauma, it is important to perform regular reassessments. ECG is a primary aid in this process as it not only assists in indicating ischaemic and traumatic myocardial damage but also identifies potentially life-threatening conditions such as pneumothorax, pneumomediastinum and pneumopericardium.

## Conclusion

This report describes a rare case of pneumopericardium with extreme ECG abnormalities after blunt chest trauma. This condition should be distinguished from other pathologies such as myocardial contusion and myocardial infarction because of the possible development of tension pneumopericardium. Early CT scanning and frequent clinical reassessments are important in the evaluation of blunt chest trauma.

## Competing interests

The author(s) declare that they have no competing interests.

## Authors' contributions

AJMK was the principal author of the paper. PHME and MAK revised and edited the whole document. All authors read and approved the final manuscript.

## Consent

Written informed consent was obtained from the patient for publication of this case report and any accompanying images. A copy of the written consent is available for review by the Editor-in-Chief of this journal.

## References

[B1] Plautz CU Perron AD Brady WJ Electrocardiographic ST-segment elevation in the trauma patient: acute myocardial infarction vs. myocardial contusion Am J Emerg Med 2005 23 510 516 10.1016/j.ajem.2004.03.014 16032622

[B2] Bansal MK Maraj S Chewaproug D Amanullah A Myocardial contusion injury: redefining the diagnostic algorithm Emerg Med J 2004 22 7 465 469 10.1136/emj.2004.015339 15983078 PMC1726836

[B3] Zajarias A Thanigaraj S Taniuchi M Acute coronary occlusion and myocardial infarction secondary to blunt chest trauma from an automobile airbag deployment J Invasive Cardiol 2006 18 E71 E73 16404791

[B4] Collins JN Cole FJ Weireter LJ Riblet JL Britt LD The usefulness of serum troponin levels in evaluating cardiac injury Am Surg 2001 67 821 825 11565757

[B5] Rowan KR Kirkpatrick AW Liu D Forkheim KE Mayo JR Nicolaou S Traumatic pneumothorax detection with thoracic US: correlation with chest radiography and CT-initial experience Radiology 2002 225 210 214 10.1148/radiol.2251011102 12355007

[B6] Wicky S Wintermark M Schnyder P Capasso P Denys A Imaging of blunt chest trauma Eur Radiol 2000 10 1524 1538 10.1007/s003300000435 11044920

[B7] Macklin CC Transport of air along sheaths of pulmonic blood vessels from alveoli to mediastinum. Clinical implications Arch Intern Med 1939 64 913 926

[B8] Mansfield PB Graham CB Beckwith JB Hall DG Sauvage LR Pneumopericardium and pneumomediastinum in infants and children J Pediatr Surg 1973 8 691 699 10.1016/0022-3468(73)90408-9 4584830

[B9] Brander L Ramsay D Dreier D Peter M Graeni R Continuous left hemidiaphragm sign revisited: a case of spontaneous pneumopericardium and literature review Heart 2002 88 5 10.1136/heart.88.4.e5 12231618 PMC1767382

[B10] Haan JM Scalea TM Tension pneumopericardium: a case report and a review of the literature Am Surg 2006 72 330 331 16676858

